# Ultrafine Particles from Residential Biomass Combustion: A Review on Experimental Data and Toxicological Response

**DOI:** 10.3390/ijms20204992

**Published:** 2019-10-09

**Authors:** Emanuela Corsini, Marina Marinovich, Roberta Vecchi

**Affiliations:** 1 Laboratory of Toxicology, ESP, Università degli Studi di Milano, 20133 Milan, Italy; emanuela.corsini@unimi.it; 2 Laboratory of Toxicology, DISFEB, Università degli Studi di Milano, 20133 Milan, Italy; marina.marinovich@unimi.it; 3 Department of Physics, Università degli Studi di Milano, and INFN-Milan, 20133 Milan, Italy

**Keywords:** biomass combustion, residential heating, ultrafine particles, chemical composition, toxicity

## Abstract

Biomass burning is considered an important source of indoor and outdoor air pollutants worldwide. Due to competitive costs and climate change sustainability compared to fossil fuels, biomass combustion for residential heating is increasing and expected to become the major source of primary particulate matter emission over the next 5–15 years. The understanding of health effects and measures necessary to reduce biomass emissions of harmful compounds is mandatory to protect public health. The intent of this review is to report available data on ultrafine particles (UFPs, i.e., particles with diameter smaller than 100 nm) emitted by residential biomass combustion and their effects on human health (in vitro and in vivo studies). Indeed, as far as we know, papers focusing specifically on UFPs originating from residential biomass combustion and their impact on human health are still lacking.

## 1. Introduction

Biomass burning is a significant air pollution source with impacts on both local and global scales, threatening air quality and visibility, and affecting the Earth’s radiative budget with effects on climate; in addition, a great concern is related to health effects [1]. Biomass burning is thus considered an important source of indoor and outdoor air pollutants worldwide.

Biomass fuels refer to any organic materials, including living plants and/or animal-based materials, produced in a renewable manner that is deliberately burned as fuel, including wood, crop residues, and animal dung.

Biomass combustion emissions, in contrast to emissions from other sources of air pollution like motor vehicles, are increasing and expected to become the major source of primary particulate matter emissions over the next 5–15 years [2]. Emissions from this source are governed by fuel type and combustion conditions. Inefficient combustion, due to relatively poor oxygen supply with the production of carbon monoxide, black carbon, and complex organic compounds, is considered a major source of indoor air pollution in the developing world [3]. Household air pollution from heating using biomass fuels is the major contributor of indoor air pollution and especially in developing countries, also cooking activities give a significant contribution. Moreover, biomass burning emissions are important contributors to outdoor air pollution, accounting for at least 10% of ambient fine and ultrafine particulate matter [4].

In recent years, in Europe the demand of biomass as a fuel for household heating has been largely increased due to being cheaper than fossil fuels and due to the implementation of climate change mitigation policies. These emissions contribute to ambient air quality especially in winter time and during nighttime when burning activities are stronger and the atmospheric mixing layer is reduced (see e.g., [5,6] and references therein). Comparing the energy demand in 2012 vs. 1990 in OECD Europe countries, a 37% increase is reported in [7] and its relative contribution changed from 8% in 1990 to 12% in 2012. With reference to the same years, in Central Europe biomass consumption showed an increase of 113% accounting for 27% to total fuel consumption in 2012. According to the inventory developed by Denier van der Gon et al. [8] for anthropogenic carbonaceous aerosol emissions in Europe, residential wood combustion was the largest contributor to organic aerosol and about half of the total fine particulate matter emissions (i.e., with aerodynamic diameter less than 2.5 µm) was ascribed to carbonaceous aerosol.

In Europe, it has been estimated that small scale domestic wood/biomass combustion will become the dominant source of fine primary particle air pollution by 2020 [9], with a contribution of 38% of total emissions [2]. The use of biomass for energy production is expected to double, whereas use of coal, gas, and oil are all expected to decrease [10].

Particles from combustion processes have a complex chemical composition, which makes it difficult to foresee the health hazards simply based on ambient concentrations, particulate matter size and composition. Experimental data suggest that biomass smoke inhalation causes lung adverse effects that are influenced by the type of fuel (e.g., peat, eucalyptus, oak), and combustion phase (e.g., flaming or smoldering), and since no striking differences in gas-phase components among different fuels under the same combustion phase were observed, particulate matter composition appears to be the key factor responsible for the different adverse lung toxicity and physiological outcomes observed following exposure to different fuels [11]. It is, therefore, important to gain knowledge on the physicochemical properties of biomass emissions to estimate effects on air quality and potential health adverse effects. In order to better understand the biological effects of the particles generated by biomass combustion, without using experimental animals, several in vitro methods at different levels of complexity have been used and proposed to verify and compare the biological activity of different biomass fuels and the effectiveness of mitigation measures [12,13,14,15,16,17,18]. Toxic effects of the emissions should be tested under the same experimental conditions, possibly including wet and dry deposition systems, if comparisons want to be made, as this will allow for ranking of the overall toxicity of the emissions. In parallel, a detailed physicochemical characterization of the emissions will allow the identification of the most important elements in determining specific biological effects, e.g., cell damage, inflammation, genotoxicity. Overall, there is a lack of evidence supporting differential toxicity of particulate matter from biomass combustion when compared with urban particulate matter from fossil fuel combustion and secondary atmospheric particles [19,20].

Among differently-sized aerosols, UFPs have drawn attention in the last years due to the little knowledge about their properties and impacts on human health. Owing to their small size, UFPs typically do not account for a significant portion of particulate matter mass but dominate in terms of number concentration; their chemical composition and spatiotemporal distribution show a high variability related to specific emission sources, distance from the source itself, and micrometeorology. These small particles are extremely dynamic in the atmosphere, undergoing to many different processes such as diffusion, coagulation, condensational growth, evaporation, and deposition. To follow the evolution of particles in ambient air and/or in laboratory experiments, on-line instrumentation with high-time resolution is needed. Many literature studies dealing with ultrafine particles (i.e., less than 100 nm in size) report on particle number concentration (PNC) but chemical composition is largely under-assessed. Despite the availability of on-line aerosol mass spectrometers with high resolution, the efficiency under 100 nm is quite low (e.g., HR-AMS has a lower limit at about 40–70 nm) and the accurate quantitative analysis has still some issues; nevertheless, these spectrometers are extremely useful for qualitative investigation [21]. It is noteworthy that the large majority of toxicological studies on ultrafine particles are still carried out in vitro exposing cells to aerosol particles previously collected and characterized as for their mass concentration and chemical composition; this is a challenging task due to the small mass associated with ultrafine particles and related analytical issues to assess UFP chemical composition.

UFPs are emitted in the atmosphere directly by natural and anthropogenic sources (primary UFPs) and/or generated by gas-to-particle processes from precursor gases (UFPs with secondary natural/anthropogenic origin). Nucleation is by far the most important new particle formation process in natural environments; it proceeds from low volatile gaseous precursors such as sulfuric acid, ammonia, amines, organics, and iodide compounds. The role of non-volatile highly oxidized molecules related to biogenic organic vapors has been recently highlighted. After the formation of thermodynamically stable clusters, particles grow due to condensation of additional vapor molecules. In uncontaminated environments, forest and biogenic marine emissions account for UFP number concentrations ranging from 10^2^ to 10^4^ particles cm^−3^ (see e.g., [22,23,24] and references therein). Peaks in UFP concentration were also related to volcanic plumes observation as reported e.g., by Schäfer et al. [25] who ascribed ultrafine particle formation to photochemical conversion of sulfur dioxide to sulfuric acid.

Combustion processes typically emit primary UFPs and gases which can promote ultrafine secondary particle formation. According to literature works, e.g., [26,27], globally the major source for urban UFPs is traffic; in particular, diesel vehicles are considered important UFP emitters because of their higher emission factors compared to gasoline engines. As reported in the literature (e.g., [28,29,30,31,32,33,34,35,36,37,38] and references therein), in addition to vehicle exhaust emissions UFPs in outdoor air can originate from a variety of sources such as industrial processes, energy production plants and incinerators, aircraft and ship emissions, construction works, tire abrasion, cooking, and domestic heating. It is noteworthy that an increase in the relative contribution from these sources is expected due to the decline of tailpipe emissions from motor vehicles as a consequence of improved technologies and more stringent regulation on emissions as, e.g., those in force in the European community. Moreover, this variety of sources has a different spatial distribution of emissions compared to road traffic and presents highly specific physicochemical properties in relation to the process which originates the ultrafine particles ([28] and references therein).

The health impact of UFPs indoor is also of great concern because in many countries the majority of people spend most of their time in confined environments. Indoor particles comprise both primary and secondary aerosols emitted by indoor sources (e.g., domestic biomass burning, vacuum cleaners, hair dryers, incense burning and candling, ironing, cooking, laser printing, and smoking in addition to emissions specifically related to workplaces) as well as outdoor aerosols infiltrated indoor [28,39,40]. A general characterization of UFP indoor sources is difficult because many indoor emissions are strictly dependent on the type of activity under investigation.

To the authors’ knowledge, a paper taking stock of the current literature on the features and impact of ultrafine particles generated by residential biomass combustion is not available yet, although UFPs are mentioned as part of previous reviews such as in [24,28]. Opposite, an increasing number of studies dealing with biomass burning assessment and toxicological outcomes are available for larger aerosol size fractions.

## 2. Characteristics of UFPs Generated by Residential Biomass Combustion

Physicochemical and optical properties of aerosols emitted by biomass burning have been extensively reported in previous papers (see e.g., [41,42]) and they will be not repeated here; instead, a collection of results dealing with UFPs will be given in the next sections. Hereafter, we will specifically refer to literature studies related to particles less than 100 nm in size emitted by biomass burning used for residential heating and many studies reporting results on other size fractions (often referred to as UFPs although having larger diameters) will not be taken into account. It is noteworthy that usually UFP measurement is performed using a variety of instruments based on different equivalent diameters (e.g., aerodynamic, electrical mobility, optical, and geometric diameters; see definitions, e.g., in [43]); literature data reported in this review refer to particles less than 100 nm in size whatever the technique used to detect them.

### 2.1. Ambient Measurements

Particles from biomass burning can originate from multiple sources such as domestic heating, cooking fires, power generation, agricultural burning as well as prescribed and wildland fires with impacts on both indoor and outdoor environments. It is well known that results on biomass burning emissions are strongly dependent on combustion appliances usage, fuel type (e.g., logs vs. pellets; hardwood vs. softwood; moisture content), burning conditions (e.g., flaming vs. smouldering), experimental conditions (e.g., air supply and dilution), and so on; therefore, particulate matter characteristics can be also quite variable.

In ambient air, biomass combustion particles are mixed with those coming from different sources and singling out their own characteristics is challenging. Corsini et al. [44] tackled the problem comparing the concentration and composition of UFPs during periods with wood burning on/off at a site where domestic biomass burning was a significant seasonal source and all other emissions were almost the same in different seasons. Interestingly, UFP mass did not show a summer-to-winter difference while some chemical components clearly pointed at the role of wood combustion for domestic heating as increased winter-to-summer concentration ratios were detected for typical tracers [45] such as levoglucosan and its isomers, K^+^, and benzo(a)pyrene. Benzo(b)fluoranthene and benzo(a)pyrene concentrations were about 11- and 5-times higher during winter than during summer, respectively; both emitted in abundance by small scale appliances burning wood and pellets as reported in [46].

Receptor modeling approaches such as Positive Matrix Factorization (PMF) (see e.g., [47,48]) retrieve the chemical profile of the biomass burning source in real-world conditions as they rely on ambient data and provide source apportionment. Currently, source apportionment studies based on size-segregated aerosol dataset comprising ultrafine particles are very few. Size-segregated results for wood burning detected in Milan (Italy) by Bernardoni et al. [49] showed three modes with geometric aerodynamic mean diameters of 36 nm, 210 nm, and 599 nm and the smallest mode apportioned only 4% of the mass in this specific source. The biomass burning source was mainly related to a local (urban) contribution and accounted for 15% of the mass in the finest particle fraction (i.e., the Aitken mode). In the size-resolved biomass profile, the ultrafine fraction gave the largest contributions to potassium, levoglucosan, and sulfate. Size-resolved submicron organic aerosol collected in Mexico City was apportioned by Ulbrich et al. [50] who found that organic aerosol by biomass burning shared a smaller fraction of ultrafine particles than organic oxygenated aerosol. As far as the contribution to carbonaceous components in the ultrafine fraction is concerned, results obtained by Xue et al. [51] in polluted California cities pointed at wood burning as the largest contributor (32–47%) to OC at the Fresno site in winter due to the widespread use of wood for domestic heating, while in Los Angeles this source became significant only during winter holidays due to the use of fireplaces; at all the investigated sites, wood burning gave only a small contribution to EC (1–3%). A source apportionment study on ultrafine particles carried out in Sacramento (California) [52] found that the wood burning source was traced mainly by pyrolized organic carbon while typical tracers such as K and Rb were below detection limits; the wood burning source gave a contribution approximately 3-times larger than any other source during the study period accounting up to 44% to the whole ultrafine fraction mass and being by far the largest contributor to OC mass (64%). Kleeman et al. [53] quantified the ultrafine particle source contributions during a wintertime severe air quality episode occurred in California and showed that wood smoke was the largest source of UFPs contributing for 3–43% to ultrafine particle mass and up to 60–80% during nighttime. A different approach to achieve ultrafine particle source apportionment in ambient air was used by Gu et al. [54] who performed a PMF analysis separately on particle number size distribution data measured at a background site and PM10 chemical composition at an urban traffic site in Germany. Their data interpretation suggested that ultrafine particles peaking at 70–80 nm in terms of number concentration were associated to stationary combustion; moreover, this source was well correlated with wood burning for residential heating detected in PM10. PMF was also successfully applied to particle number concentration data by Krecl et al. [55] who identified a local residential wood combustion source characterized by a peak around 70 nm; PNC for the ultrafine fraction accounted for 77% of the average number concentration but residential wood burning provided the highest contribution to volume concentration for 80 < d_p_ < 380 nm while local traffic dominated the number concentration for particles with diameter smaller than 80 nm.

### 2.2. Laboratory Scale Experiments

Results from laboratory scale experiments are also affected by a wide variability because each study has its own specificity in terms of burning overall conditions, fuel type, and measurement techniques. Johansson et al. [56] compared the emissions from old and modern residential boilers fired with wood logs and wood pellets; they found that UFPs mass and number concentrations were higher with poorer burning conditions and this was ascribed to the role played by carbonaceous particles. In contrast, Tissari et al. [57] reported that improved combustion conditions led to a particle size decrease and number concentration increase; moreover, in the particle number distributions, ultrafine particles appeared to be related to the released ash forming material in combustion and the variations in particle size were likely determined by the quantity of condensed organic vapor in the flue gas. UFP composition from conventional masonry heater burning beech wood was dominated by K, S, and Zn, and other contributors were C, Ca, Fe, Mg, Cl, P, and Na. Similarly, Torvela et al. [58] detected UFPs mainly composed of ash material in the emissions of a research combustion unit burning wood chips; indeed, they measured 13 nm sized zinc oxide particles acting as condensation nuclei for inorganic and organic material and having an outer layer made of alkali salts. Water-soluble components were measured by Park et al. [59] in size-segregated samples collected during the combustion of 10 different biomass fuels (mainly agricultural and forest residues); UFPs showed higher amounts of organic matter, potassium, sulfate, and chloride than modes characterized by a larger size. Using a combustion cycle simulating the real-world usage of a commercial pellet and a wood stove, Ozgen et al. [46] studied ultrafine particle emissions burning fir and beech logs and pellets. In this experiment, total carbon concentrations dominated UFP composition in samples from wood log combustion and potassium was significant in particles emitted by pellet burning. Moreover, PAHs were more abundant in wood samples than in pellets samples. Anhydrosugars (i.e., levoglucosan, mannosan, and galactosan) were found only in wood stove UFP samples with beech combustion. The lack of these compounds in samples from pellet combustion was ascribed to the fact that levoglucosan formation typically occurs at low temperatures, which are not usual in automatic appliances. Grilli et al. [60] analyzed biomass and diesel particles in samples characterized by aggregates less than 50 nm in size; spruce pellets burned in a modern automatic 25 kW boiler were the investigated biomass samples. They found that diesel samples had a higher amount of metals apart from Mn and K which were higher in biomass samples; PAHs were much more abundant in diesel samples (about 10-fold) and the largest PAHs contributors in biomass samples were fluoranthene and pyrene. A detailed study on the PAHs content of UFPs was carried out by Kleeman et al. [61] who analyzed organic molecules in the emissions from hardwoods and softwoods burned in a residential fireplace. In these samples, light PAHs (e.g., fluoranthene and pyrene) dominated PAHs emissions and the largest contributions to the organic content were given by levoglucosan, coniferylaldehyde (with high concentrations in pine some emissions), and sinapic aldehyde and syrinealdehyde higher in oak and eucalyptus wood smoke.

In industrialized countries, people spend the large majority of their time in indoor environments; therefore, exposure of individuals to indoor sources is an issue. It is worth noting that ultrafine and fine particles can easily penetrate to indoor air so that outdoor pollution (e.g., pollutants emitted by traffic) can also have an impact indoor and the relative contributions of different sources is not easily distinguishable. Wood burning appliances and cooking wood stoves are significant sources of UFPs indoor. Indeed, although modern wood stoves are sealed off from the room air, potential emissions occur while opening the fire chamber to refuel the stove or due to pressurization effects in the flue gas. Salthammer et al. [62] monitored indoor air quality before, during and after operation of seven wood burning fire-places in private homes in Germany. Peak number concentration registered for particles in the size range 5.6–560 nm was in the range 10^3^–10^5^ particles cm^−3^ with the largest contribution due to particles less than 100 nm in size. Similarly, Carvalho et al. [63] selected seven Danish residential buildings equipped with certified wood stoves (i.e., maximum energy efficiency with minimum particle emission); they observed notable UFPs emission indoor and the highest number concentration was recorded when the user started the fire in the stove or during wood refuel. In Southern Italy, de Gennaro et al. [64] monitored indoor air quality in six houses where heating systems based on wood burning stoves or fireplaces were operated. Results showed that biomass burning in fireplaces generated a unimodal distribution peaking between 70 and 90 nm, and typically UFP concentration was lower in large and well-ventilated rooms.

## 3. Interactions between Particulate Matter Exposure from Biomass and Human Health: In Vivo and In Vitro Effects

The World Health Organization estimates that household air pollution, mainly attributable to biomass combustion generated particulate matter, accounts for approximately 3.8 million deaths per year and an additional 4.1 million from outdoor air pollution [65]. Where for children the greatest burden of indoor air pollution-related premature death is pneumonia, for adults is cardiovascular diseases, including stroke, ischemic heart disease, with chronic obstructive pulmonary diseases and lung cancer important causes of premature death especially in women. Regions where these risks are greater include Sub-Saharan Africa and Southeast Asia [3]. In Europe, the current contribution of biomass smoke to premature mortality amounts to at least 40,000 deaths per year [2].

Moreover, not included in the burden of disease estimates for indoor air pollution, there is substantial evidence of associations between indoor air pollution and asthma exacerbation and tuberculosis [66,67]. For example, the use of open fires for cooking has been associated with an increased risk of symptoms of asthma and of asthma diagnosis in children, where no evidence of an association between the use of gas as a cooking fuel and either asthma symptoms or asthma diagnosis were found [68].

The aim of toxicology studies is to identify possible health effects and causally link them to specific exposure in population and sensitive or susceptible populations, and possibly to identify the exposure threshold level needed to induce adverse health effects. For air pollution, the main route of exposure is the respiratory tract, while adverse effects may be local or systemic, with oxidative stress and inflammation playing a central role in the development of both adverse respiratory and cardiovascular effects [68]. In addition to the inhalation exposure, the dermal route of exposure must be properly taken into account; as an example, we can recall the correlation between scrotum tumor and exposure to soot in the chimney sweepers of 19th century in England [69].

### Mode of Action

The respiratory tract is designed, both anatomically and functionally, so that the air that reaches the respiratory region is the cleanest possible: nasal hairs, nasal turbinates, cilia of the bronchial epithelium, the sneeze and cough reflexes, etc., all contribute to this filtering process [70]. Despite this, due to an unbalanced ratio between particle deposition and clearance efficacy, inhaled particles, including the ones generated by biomass combustion, can reach the deep lung. Particle deposition is strictly dependent upon particle size distribution (aerodynamic diameter), hygroscopicity, and solubility. Whereas the size determines how deep particles can penetrate the lung, hygroscopicity is related to the possibility of absorbing or exhaling water vapor, meaning that particles can get larger or smaller in size upon entering into the airway, with the consequent modification in the deposition pattern compared to what was initially expected depending on the settings [70,71]. In addition to particles size, breathing conditions are also important in determining the amount of particle deposited: increase in number of breaths, due for example to increased physical activity, leads to greater deposition.

**Figure 1:**
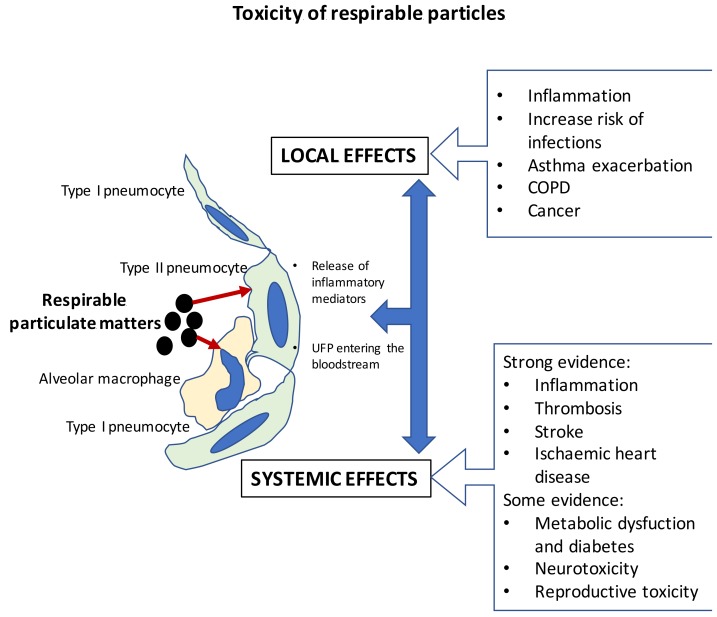
Possible adverse effects of exposure to respirable particulate matter. Inhaled particles, including the ones generated by biomass combustion, can interact with alveolar macrophages and pneumocytes resulting in cell activation, oxidative stress, glutathione depletion, DNA damage, cell death, and release of inflammatory mediators, which mediate local inflammation and, if spilled over, the systemic circulation, can contribute to adverse effects in other organs. Ultrafine particles can also enter the bloodstream, further contributing to systemic effects. Overall, the potential adverse effects of biomass smoke is expected to be similar to the ones described for tobacco smoke and ambient particulate matter, with the evidence, however, that the effects strictly depend on the type of biomass and combustion methods.

Once the particles get in contact with the airways and alveoli, they start a series of events resulting in several possible adverse effects as outlined in Figure 1 and mentioned above. Central in lung toxicity is the uptake by alveolar macrophages and pneumocytes, where particularly macrophages have a key role of scavenger in vivo, as these cells can recognize and eliminate ‘non-self’ materials [72]. The contact of particles with lung lining fluid and tissue results in antioxidant depletion, oxidative stress, and particle uptake leading to cell activation and release of inflammatory mediators, which may result in local and systemic toxicity.

Particle size and composition are directly linked to their potential to cause health hazards, as they influence lung deposition and fate, with nano-sized particles accumulating in the cells at a faster rate and promoting the secretion of Th1-specific molecule signals (e.g., IFN-γ, IL-12) compared to larger particles [73]. The different cellular responses according to the size of the particles can have a great importance in determining subsequent pathological effects. Aside from particle size, composition and surface properties will also profoundly affect the biological outcomes. For example, data indicate that the allergic responses tend to be more associated with the organic fraction of particles, whereas the inflammatory reactions seem to be more associated with metals and endotoxin [16].

Combustion particles from biomass sources may differently impact on lung cells according to the various pathways and produce different toxic effects [15]. Biomass smoke toxicity is related to the particulate matter chemical composition, which reflects the quality of the combustion and the fuel in particular [74], while particle size influences the deposition inside the respiratory tract as well as the mechanisms of cellular uptake [73].

**Figure 2:**
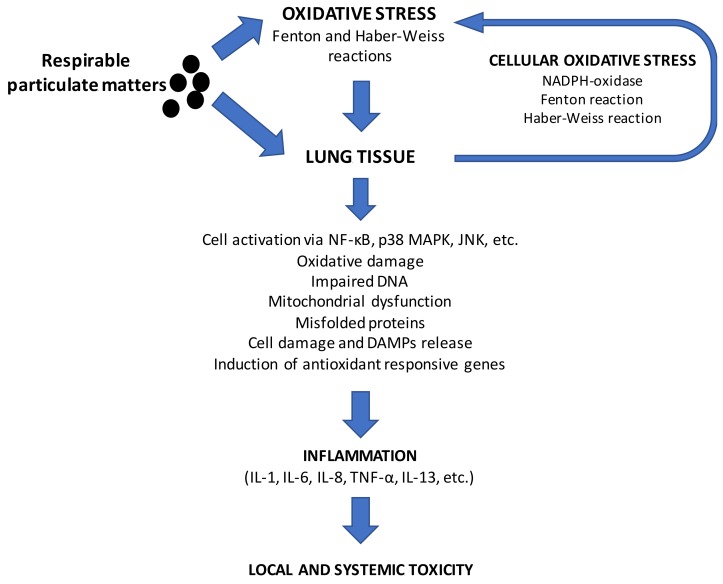
Biological pathways linking combustion particle-induced oxidative stress and adverse health effects. Respirable particulate matter, which includes UFPs, has a highly adsorptive carbon core that can deliver within the lung redox-active metals, polyaromatic hydrocarbons, and quinones. Oxidative stress has an instrumental role in the toxicity of combustion particles, and their oxidative potential has been used as an effective exposure metric, providing a biologically-relevant index of particulate activity [75]. The oxidative potential is typically assessed by the capacity of the particles to deplete physiologically relevant antioxidants like ascorbate or glutathione. Thus, reactive oxygen species can be generated directly by the particle surface in short times but also as secondary process due to cellular response. Oxidative damage, depletion of antioxidants is followed by upregulation of redox-sensitive transcription factors (NF-κB and AP-1) and their associated upstream stress-related mitogen-activated protein kinases (p38, JNK) in lung cells, which trigger the expression of proinflammatory cytokines and induce inflammation. In addition, the alteration of the cellular redox status together with the presence of genotoxic compounds adsorbed on particles, can cause DNA damage. All of this together leads to sequela of cellular adverse events, resulting in local and systemic toxicity.

A key role in the mechanism by which combustion particles, including UFPs, cause adverse health effects is played by the oxidative stress in the airways and alveoli; it leads to the activation of alveolar macrophages and pneumocytes, resulting in accumulation of inflammatory cells from the circulation (Figure 2). This local lung inflammatory reaction can spill over into systemic circulation and contribute to cardiovascular system and reproductive adverse effects, including low birth weight and cognitive impairment [3].

Alveolar macrophages laden with carbon particles have been shown to have defective responses, including decreased phagocytosis, type 1 cytokine and antimicrobial peptides production following pathogen challenge, contributing to increased risk of respiratory tract infections [65]. While inhibiting interferon-γ production, particulate matter stimulates innate lymphoid cells to produce high levels of interleukin (IL)-5 and IL-13, inducing airway hyper-responsiveness and asthma in genetically predisposed individuals [76]. These findings highlight potential mechanisms by which innate lymphoid cells react to air pollution that increase the susceptibility to infections and allergies.

The oxidative stress and the generation of reactive oxygen species, together with the presence of genotoxic compounds absorbed on particles—e.g., polycyclic aromatic hydrocarbons and metals—can account for DNA damage, providing a biological rationale for the risk of lung cancer associated with exposure to particulate matter [77,78]. The meta-relative risk for lung cancer associated with ambient air PM2.5 was 1.09 (95% CI: 1.04, 1.14), estimates for adenocarcinoma associated with PM2.5 and PM10 were 1.40 (95% CI: 1.07, 1.83) and 1.29 (95% CI: 1.02, 1.63), respectively [77]. Outdoor air pollution is classified as Group 1 carcinogen by the International Agency for Research on Cancer (IARC), while biomass fuel (primarily wood), indoor emissions from household combustion, is designated a Group 2A carcinogen [79].

Corsini et al. [12,13] carried out an in vitro study aiming at investigating the pro-inflammatory and genotoxic effects of UFP generated by wood combustion (soft vs. hard wood; pellets vs. log woods) using the human THP-1 and A549 cell lines. On a mass basis, they found that UFPs had a biological reactivity comparable to that of PM10, arguing against a higher biological activity of UFP compared with other categories of combustion-derived particle. This observation agrees with other studies demonstrating the coarse particulate matter has an inflammatory potential like fine particles on an equal mass basis [16]. In addition, regarding the inflammatory effect, a role of levoglucosan in the production of interleukin-8 (IL-8) was demonstrated, while genotoxicity was related to the presence of some elements, such as Al, Fe, and polycyclic aromatic hydrocarbons [44]. Among the pro-inflammatory mediators induced by particles, the measurement of IL-8 is of relevance as this chemokine is a neutrophil attractor, and an influx of neutrophils may lead to a sustained inflammation and possible tissue damage [80,81]. Thus, increased IL-8 can contribute to the risk of disease development as well as exacerbate existing conditions by inducing an immunologically active state.

The significant differences in biological reactivity in relation to the wood type (soft vs. hard wood; pellet vs. wood) observed are interesting and relevant for a possible emission control. Overall, pellet wood should be preferred to wood logs as a fuel for domestic heating in terms of emissions; moreover, literature results show that hardwood pellet is generating biologically less reactive UFP than softwood pellet [44]. In the work by Corsini et al. [13,44], both cell lines responded to UFP producing IL-8, with UFPs produced by log wood combustion being more active compared to UFPs emitted by pellet combustion. UFP-induced IL-8 could be reduced by SB203580, indicating a role of p38MAPK activation in IL-8 production. The higher activity in THP-1 of UFPs produced by beech log wood combustion was not due to higher uptake or endotoxin contamination; the latter was demonstrated by the use of polymyxin B to sequester endotoxin [44]. A possible explanation can be given by the observation of qualitatively different protein corona adsorption profiles with fewer proteins bound to samples collected during beech combustion UFPs compared to those emitted by conifer burning or DEP; this can promote higher intracellular availability of bioactive components—i.e., levoglucosan and galactosan—toward which THP-1 cells were more responsive compared to A549 cells.

Particles emitted during wood combustion could be enriched of combustion by-products, including polycyclic aromatic hydrocarbons, ions, heavy metals, and other pyrolytic products, which can all contribute to DNA damage [82]. In a study aimed at characterizing the genotoxic potential of ultrafine particles produced by the combustion of two widespread types of wood (beech and fir) in state-of-the-art commercial pellet and logwood room-heaters on human type II alveolar cells (A549 cell lines), all the UFPs after 24 h of treatment elicited DNA damage, with the fir log wood UFPs determining the most significant increase of DNA damage compared to beech wood or pellet, as assessed by Comet assay [82]. In term of composition, UFPs generated from batch-wise room heater showed definitely a much higher polycyclic aromatic hydrocarbons fraction than the UFPs from pellet combustion [82], which is important in the choice of heater systems with lowest impact.

These data further confirm the importance of fuel type and burning conditions in the biological reactivity of combustion-generated particles, and highlight how the size of particles could not account for variation in biological reactivity among particles but rather the composition seems to be the driving force to trigger inflammation.

Size, surface area, solubility, and particle composition appear all to be critical in the different biological effects of wood smoke particles. Overall, in vivo and in vitro experiments demonstrate that wood smoke particles can induce inflammatory responses, cytotoxicity, genotoxicity, oxidative stress, and immunosuppressive effects [2].

Differences in the properties of biomass burning particles indoor in relation to the overall combustion features and fuel type as well as various physicochemical characteristics of outdoor particulate matter resulting from seasonal differences can profoundly affect the interactions with biological systems [83]. Corsini et al. [13,44] and Marabini et al. [82] demonstrated that UFP collected in winter or summer have a different biological behavior: those sampled in summer are more pro-inflammatory, while winter ones are more genotoxic. This can be explained by a different sensitivity to the different chemical components found in UFPs depending on season—e.g., polycyclic aromatic hydrocarbons are higher in winter time—while endotoxin is higher in summer time resulting in a different cellular reactivity [44,84,85].

## 4. Conclusions

Many studies have shown that national, state, and local regulatory actions taken to reduce emissions resulted in decreased outdoor pollution and improved air quality; moreover, associations between ambient air quality and adverse health outcomes to indirectly infer health benefits of regulatory actions have been demonstrated [83].

Despite decades of effort to promote cleaner cooking and heating technologies based on biomass fuels, the displacement of polluting technologies has progressed slowly [86]. Biomass combustion emissions are increasing also in developed countries, and are expected to become the major source of primary particulate matter emission over the next 5–15 years. There is increasing evidence about the role of wood log stoves in generating particles more biologically active compared to pellet stoves; in addition, it is clear that different wood essences generate particles with different composition and biological activity. Therefore, a strategic area of health research on ambient air and particulate matter emissions is the development and use of clean combustion approaches and emission control technologies together with proper education of users.

Further studies are needed to clarify the role of combustion conditions and biomass fuels in particle size distribution and composition, and to understand the molecular mechanisms behind the harmful effects observed. A shift to clean fuel alternatives are desirable to reduce exposure to biomass combustion products.

UFPs large specific surface area makes them potentially more harmful than other particulate matter fractions because it facilitates the absorption of toxic components and the small size of these particles promotes their transport to different regions of the body. Detailed physicochemical characterization of ultrafine particles from biomass burning aiming at the identification of possible biological effects and underlying mechanisms is mandatory but still very challenging due to the little mass contribution given by UFPs that claims for advanced analytical approaches. In addition, the identification of UFP sources in indoor and outdoor air—like domestic biomass burning here discussed—is required to improve air quality and to implement targeted strategies for the abatement of this particle fraction leading to healthy environments; this is also very difficult because of the above-mentioned challenging measurements needed but also because UFPs are emitted by a variety of sources.

Finally, better knowledge about physicochemical properties of ultrafine particles generated by biomass combustion will be helpful in filling the gap about the relationship with potential toxicity and biological outcomes.

## Abbreviations

IL	interleukin
PM	particulate matter
UFP	ultrafine particles
